# Ambrisentan Retains Its Pro‐Autophagic Activity on Human Pulmonary Artery Endothelial Cells Exposed to Hypoxia in an In Vitro Model Mimicking Diabetes

**DOI:** 10.1111/jcmm.70528

**Published:** 2025-04-09

**Authors:** Manuela Cabiati, Filippo Biondi, Sandra Ghelardoni, Valentina Casieri, Vincenzo Lionetti, Agnese Sgalippa, Silvia Del Ry, Rosalinda Madonna

**Affiliations:** ^1^ Laboratory of Biochemistry and Molecular Biology Institute of Clinical Physiology, CNR Pisa Italy; ^2^ Department of Pathology, Cardiology Division University of Pisa Pisa Italy; ^3^ Department of Pathology, Laboratory of Biochemistry University of Pisa Pisa Italy; ^4^ Unit of Translational Critical Care Medicine, Laboratory of Basic and Applied Medical Sciences Interdisciplinary Research Center “Health Science,” Scuola Superiore Sant'Anna Pisa Italy

**Keywords:** ambrisentan, autophagy, endothelin receptor antagonist, high glucose, hyperosmolar stress, miR124‐3p, miR191‐3p

## Abstract

Cardiovascular comorbidities are associated with reduced treatment response in group 1 pulmonary arterial hypertension (PAH). This may result from misdiagnosis of group 2 PH, but it can also be explained as the loss of ability of pulmonary endothelial cells to respond to specific antiremodeling drugs. We evaluated the effects of high glucose (HG) and hyperosmolar stress (high mannitol, HM) on the response of human pulmonary artery endothelial cells (hPAECs) to ambrisentan (AMB), focusing on autophagy, viability, apoptosis and several microRNAs involved in pulmonary arterial remodelling. hPAECs were incubated with 30.5 mM HG or 25 mM HM, with/without 0.02 nM AMB in normoxia (Nx) or hypoxia (Hx) for 24 h. Hx reduced cell survival (*p* = 0.03) and autophagy (*p* = 0.02), an effect mimicked by HG and HM only in Nx. In Nx and Hx, AMB reverted the effect of HG, but not HM on autophagy, almost completely or partially, respectively. Compared to Nx, Hx increased the antiapoptotic miR124‐3p in vehicle‐treated hPAEC (*p* = 0.002), and induced an opposite effect on antiapoptotic and proliferative miR191‐3p. In Nx, AMB induced miR124‐3p in HG‐ (*p* = 0.04 vs. HG+A_Nx) and HM‐treated (*p* < 0.0001 vs. HM+AMB_Nx) hPAECs, and miR191‐3p in HM‐treated hPAECs (*p* = 0.03). In H, A induced a similar effect on miR124‐3p in hPAEC exposed to AMB+HM (*p* = 0.02). In hPAEC exposed to Hx, AMB retains its pro‐autophagic effects in an in vitro model mimicking diabetes. miR124‐3p and, to a lesser extent, miR191‐3p may act as biomarkers of disease and treatment response to specific drugs in patients with PAH and diabetes.

## 1 Introduction

Cardiac or metabolic comorbidities, such as arterial hypertension, diabetes and dyslipidemia, affect the prognosis and response to treatment of PAH, specifically undermining the efficacy and/or tolerability of combination therapy, the mainstay for the comorbidity‐free PAH [1, 2]. This issue was first addressed in a post hoc analysis of patients excluded from the Ambrisentan and Tadalafil in Patients with Pulmonary Arterial Hypertension (AMBITION) trial due to having more than three cardiovascular or metabolic comorbidities [3]. Registries have since confirmed that pulmonary vasodilators generally have limited efficacy and reduced tolerability in the comorbid setting [4, 5, 6, 7, 8, 9], irrespective of the number and type of cardiopulmonary comorbidities [5, 6]. In particular, these limitations strongly apply to the use of endothelin receptor antagonists (ERAs) and prostacyclins in mono‐ or combination therapies [3, 5, 9], while, conversely, monotherapies with phosphodiesterase type 5 inhibitors/soluble guanylate cyclase stimulators have been found to better retain their efficacy and tolerability [3, 9, 10].

Given the clinical and diagnostic complexity of PAH, comorbid cases may underlie an unrecognised post‐capillary component, so their resistance to treatment may be a matter of misclassification [11]. Thus, a proportion of comorbid PAH may rather be heart failure (HF) patients with an inapparent post‐capillary PAH at rest. Of note, little, if any, effort has been made to investigate whether any specific biological feature of PAH with metabolic comorbidities including diabetes may mediate resistance to pulmonary vasodilators and their anti‐remodelling effect. Diabetes, therefore, mimicked in vitro by high glucose, can interfere with the response of pulmonary endothelial cells to PAH‐specific drugs, making them resistant to their vasodilatory and anti‐remodelling effect.

Specific microRNAs (miRNAs/miRs) have emerged as key players in PAH, playing a crucial role in its development and progression [12]. Identification of circulating miRNAs in patients with PAH may provide novel diagnostic, prognostic, and therapeutic opportunities. Specifically [13], it has been reported that miR‐191 expression is significantly increased in all PAH patients, especially in those with diabetes and it increases further as PAH severity progresses. miR‐124 [14] plays a key role in controlling the metabolic, proliferative and proinflammatory phenotype of PAH. miR‐146a is a biomarker of oxidative stress and promotes vascular smooth muscle cell proliferation and vascular neointimal hyperplasia [15]. miR‐7110‐3p has a proliferative and anti‐apoptotic effect on pulmonary arterial smooth muscle cells. Finally, miR‐193‐3p has a proliferative and anti‐apoptotic effect on pulmonary arterial smooth muscle cells [16].

In this exploratory in vitro study, we addressed the impact of hyperosmolar stress induced by high glucose or high mannitol concentration, and/or hypoxia on the response of human pulmonary artery endothelial cells (hPAECs) to the endothelin receptor antagonist (ERA) ambrisentan (AMB) in terms of viability, apoptotic and autophagic activity, and expression of miR191‐3p and miR124‐3p. ERAs possess anti‐remodelling properties which have been demonstrated in animal models [17, 18]. Autophagy and the expression of miR191‐3p and miR124‐3p were selected as biomarkers due to their emerging role in PAH pathogenesis, particularly in pulmonary artery remodelling [16, 19, 20, 21].

## 2 Materials and Methods

Ambrisentan, D‐glucose and D‐mannitol were purchased from Sigma Aldrich (St Louis, Missouri). Human pulmonary artery endothelial cells (hPAECs) were purchased from Cascade Biologicals (Portland, Oregon). Ambrisentan powder was dissolved in DMSO at a concentration of 2 mg/mL, equivalent to a 5.4 mM working solution. D‐glucose and D‐mannitol were dissolved in culture medium to form a working solution of 500 mM. Final concentrations of each stimulus (ambrisentan, glucose and mannitol) were obtained by diluting the respective working solutions in the culture medium. The vehicle consists of culture medium with DMSO at a percentage much lower than 0.001%.

### 2.1 Cell Cultures and Treatments

The hPAECs (Cat. no C‐008‐5C, Lot#6C0189, Cascades Biologicals, USA) were maintained in Medium 231 with low serum growth supplement (Life technologies, USA). The cells were used in the study from passage 3–5. Hypoxia was induced by exposing the cells to 4% O_2_ with 4% CO_2_ and 92% N_2_ for 24 h at 37°C. Cells were incubated with control d‐glucose concentration (Vehicle, 5.5 mmol/L, control and 285 mOsm/L), high glucose (HG: 30.5 mmol/L and 385 mOsm/L), high mannitol (HM: 5.5 mmol/L glucose +25 mmol/L and 385 mOsm/L), for 24 or 48 h with/without ambrisentan (AMB) (0.02 nM) in normoxia (Nx) or hypoxia (Hx). At the end of treatments, cells were harvested for RNA isolation, or incubated with specific dyes for MTT and autophagy assays.

#### 2.1.1 Measurement of Cell Viability

The cytotoxic effect of HG and HM with or without AMB was determined using a 3‐(4,5‐Dimethylthiazol‐2‐yl)‐2,5‐diphenyltetrazolium bromide assay (MTT, Sigma). hPAECs were harvested, diluted to 2 × 10^4^ cells per 100 μL, and seeded in 96 well plates. hPAECs were treated with HG, HM or vehicle (Con, DMSO) or AMB (0.02 or 0.2 nM), in single or cotreatment, under hypoxia or normoxia conditions for 48 h. To assess AMB's toxicity, hPAECs were treated with AMB in a concentration range from 0.02 to 5000 nM, for 48 h. At the end of each incubation, MTT test was performed. Briefly, MTT (0.5 mg/mL) was added to the medium, and after 4 h an SDS–HCl solution (0.05 mg/mL) was used to solubilise the formed formazan salt. The absorbance of the solution was read at 570 nm after 18 h in a microplate reader (Bio‐Rad Laboratories, Hercules, USA). Cytotoxicity was also assessed by measuring lactate dehydrogenase (LDH) activity. Briefly, LDH activity was measured in culture media upon treatments by LDH Cytotoxicity Assay Kit (Abcam cat#102526) according to the manufacturers' protocols, which are based on the reduction of a tetrazolium salt to a formazan dye. Results were expressed as absorbance arbitrary units of LDH activity read at 570 nm. All samples were tested in 8 replicates. The results were verified with a positive control provided by the kit.

#### 2.1.2 Autophagy Detection by Immunofluorescence

The effect of HG, HM with or without AMB in Hx and Nx conditions on the autophagy of live hPAECs was detected by the Autophagy Detection Kit (Abcam ab139484, Cambridge, UK) in accordance with the vendor's protocol (*n* = 3 independent experiments). hPAECs were plated at 2 × 10^5^ cells per well in a 6‐well chamber slides, grown overnight, then exposed to Nx or Hx and treated with HG, HM or AMB in single or cotreatment for 24 h as follows. After blocking with 1% BSA (Sigma‐Aldrich) for 30 min at room temperature, fluorescent dyes for nuclei staining and autophagy detection were added and incubated for 30 min at room temperature. After three washes using PBS, the autophagic vacuoles determining the green‐fluorescent punctate pattern were observed under fluorescence microscopy. The percentage of green fluorescence intensity was assessed by image analysis to determine the degree of autophagy.

### 2.2 Autophagy and Apoptosis Detection by Immunoblotting

The effects of HG, HM with or without ambrisentan in hypoxia and normoxia conditions on the expression of specific markers for autophagy (LC3‐II) and apoptosis (cleaved caspase‐3) were examined in hPAECs treated with HG, HM or vehicle (DMSO) or AMB (0.02 nM) under Hx or Nx conditions for 24 h. In parallel experiments, cells were treated with the autophagy inhibitor chloroquine (CL). Total proteins were isolated in an ice‐cold Radio Immuno Precipitation Assay (RIPA) buffer, separated under reducing conditions and electroblotted onto polyvinylidene fluoride membrane (Immobilon‐P, Millipore, Bedford, MA). After blocking, the membranes were treated for the immunoreactions as detailed in Mattii et al. [22]. Equal loading/equal protein transfers were verified by normalising to GAPDH or β‐actin.

### 2.3 Transcriptional Analyses

#### 2.3.1 RNA Extraction and Real‐Time PCR Experiments

Total RNA was extracted from hPEACs (*n* = 3 independent experiments) cell culture treated with HG, HM or vehicle (DMSO) or ambrisentan (0.02 nM), under hypoxia or normoxia conditions for 24 h, by a dedicated kit (RNeasy Plus Micro Kit, Qiagen SpA, Milano, Italy) optimised to purify total RNA from small amounts of cells (< 5 × 10^5^) as reported in our previous work [23]. Cells treated with CL were also collected, and RNA was similarly extracted. Briefly, after re‐suspensions, hPAECs were first lysed and homogenised in highly denaturing guanidine‐isothiocyanate‐containing buffer RLT Plus, which immediately inactivated RNases to ensure isolation of intact RNA. Then, the samples were passed through a gDNA Eliminator spin column. Ethanol was added to the flow‐through to provide appropriate binding conditions for RNA, and then the samples were applied to a silica‐based membrane (RNeasy MinElute spin column) and speeded on microcentrifuge at 12000 RPM for 30 s; specific buffers allowed RNA to bind to the RNeasy silica membrane and contaminants were efficiently washed away. High‐quality RNA was then eluted in RNAse‐free water without additional DNase digestion.

The total RNA sample concentration was determined by measuring the absorbance at 260 and 280 nm (NanoDrop Thermofisher, Waltham, MA, USA) and calculated using the Beer–Lambert law (expected values between 1.8 and 2.1). The total RNA was then reverse‐transcribed in first‐strand cDNA by the Mir‐X miRNA first strand synthesis kit (Takara BIO, USA). The miRNA expressions were determined by Real‐Time PCR in the Bio‐Rad C1000 TM thermal cycler (CFX‐96 Real‐Time PCR detection systems, Bio‐Rad) and monitored with a third‐generation fluorophore, EvaGreen/Sybrgreen (SsoFAST EvaGreen/Sybrgreen Supermix, Bio‐Rad). Mature miRNA sequences used as forward primers for miRNA detection were downloaded from the miRBase database (www.mirbase.org) and synthesised by the Merck‐Sigma company (Milan, I) (Table S1). To assess product specificity, amplicons were systematically checked by melting curve analysis. Melting curves were generated from 65°C to 95°C with increments of 0.5°C/cycle. The MIQE Guidelines [24] for a correct and reproducible real‐time PCR experiment were followed (Table S1).

### 2.4 Statistical Analysis

For cell viability and autophagy detection, results are reported as mean ± SD and represent data from a minimum of three independent experiments, if not indicated otherwise in the legend of the figures. Differences between groups were analysed by one‐way ANOVA followed by Dunnett's or Tukey's test for multiple comparisons. Parametric student's *t*‐test or non‐parametric Mann‐Whitney test for unpaired data were used to evaluate single comparisons between different experimental groups.

The ∆∆Ct method was used for biomolecular analysis to quantify miRNA levels, normalising their relative expression with U6 snRNA transcripts (TaKaRa Bio USA Inc.). For the multiple comparisons, Fisher's test was used after the analysis of variance (ANOVA); the results were expressed as mean ± SEM, and the *p*‐value was considered significant when < 0.05. When the expression values were not normally distributed, the data logarithmic transformation was used for statistical analysis. The statistical analysis of the results was carried out through both Stat‐View 5.0.1 software released for Windows Statistical (1992–98, SAS Institute Inc., SAS Campus Drive, Cary, NC, USA) and GraphPad Prism version 6.0 for Windows (GraphPad Software, San Diego, CA, USA).

## 3 Results

### 3.1 Ambrisentan and High Glucose Do Not Further Impact the Cytotoxic Effect of Hypoxia in hPAECs

**FIGURE 1:**
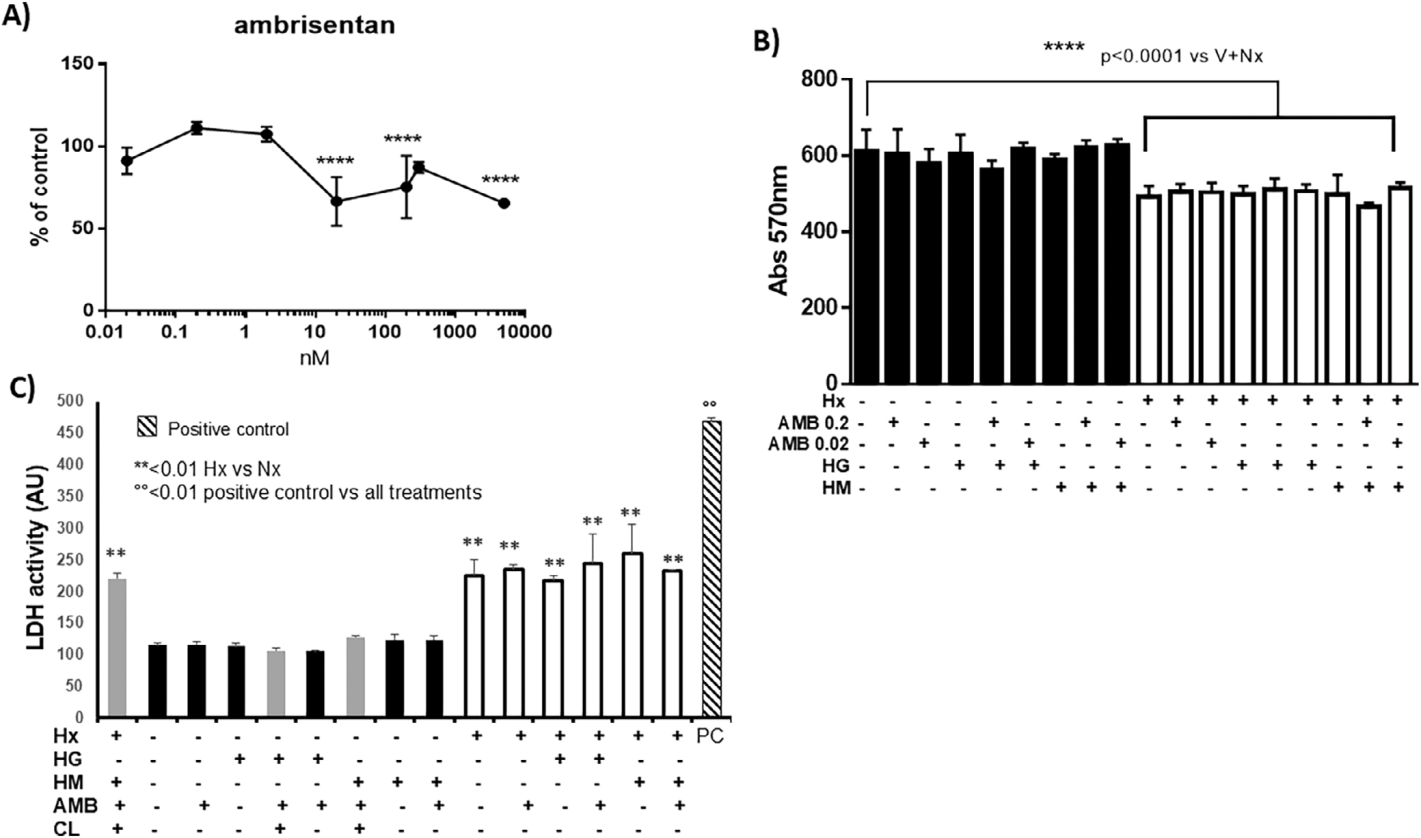
Ambrisentan and high glucose do not impact the cytotoxic effect of hypoxia in hPAECs. (A) The line graph represents the impact of increasing concentrations (0.02–5000 nM) of ambrisentan on the cell viability of human pulmonary artery endothelial cells (hPAEC) by the MTT assay. Statistical analysis was performed by one‐way ANOVA followed by Dunnett's multiple comparison: ****p < 0.0001 versus control (Con, DMSO). (B, C) hPAECs were treated with control D‐glucose (vehicle, V), 0.02 or 0.2 nM ambrisentan (A0.02 and A0.2, respectively), high glucose (HG), high mannitol (HM), alone or in combination as illustrated in graph, for 48 h, in conditions of normoxia (Nx) and hypoxia (Hx). Cell viability was determined by MTT assay (B) and LDH activity assay (C) in which treatments were repeated in the presence or absence of autophagy inhibitor chloroquine (CL). Statistical analysis was performed by one‐way ANOVA followed by Tukey's multiple comparison: (B) ****p < 0.0001 vs. V+Nx (control in normoxia, DMSO). (C) **p < 0.01 Hx vs. Nx. °°*p* < 0.01 all treatments vs. positive control (PC).

We first evaluated the toxicity of increasing concentrations of AMB (from 0.02 nM to 5000 nM) and then the non‐cytotoxic concentration of AMB (0.02 or 0.2 nM), in combination with hyperosmolar stress to assess, by MTT test which measures succinate dehydrogenase activity or by LDH activity assay in cell culture medium, whether these conditions exerted a further impact on hypoxia‐induced cytotoxicity. AMB significantly reduced hPAEC cell viability in Nx only starting from 20 nM (Figure 1A). Compared to Nx, 24 h Hx induced a significant reduction in hPAEC cell viability in all treatment groups (Figure 1B). Similar results were obtained by LDH activity assay where an increase of LDH was reported in hypoxic conditions which were not worsened by AMB or hyperosmolar treatments (Figure 1C). AMB (0.02 or 0.2 nM), HG, and/or HM did not exhibit any additional cytotoxic effect in hypoxic conditions when compared to the hypoxic control group (V+Hx), concluding that hyperosmolarity induced by HG or HM did not alter mitochondrial function. These results suggest that hypoxia affected hPAEC viability, but at the concentrations tested, treatments including the hyperosmolar stress did not further impact on hypoxic cytotoxicity.

### 3.2 Ambrisentan Restores Autophagy in hPAECs Treated With High Glucose Under Hypoxic and Normoxic Conditions

**FIGURE 2:**
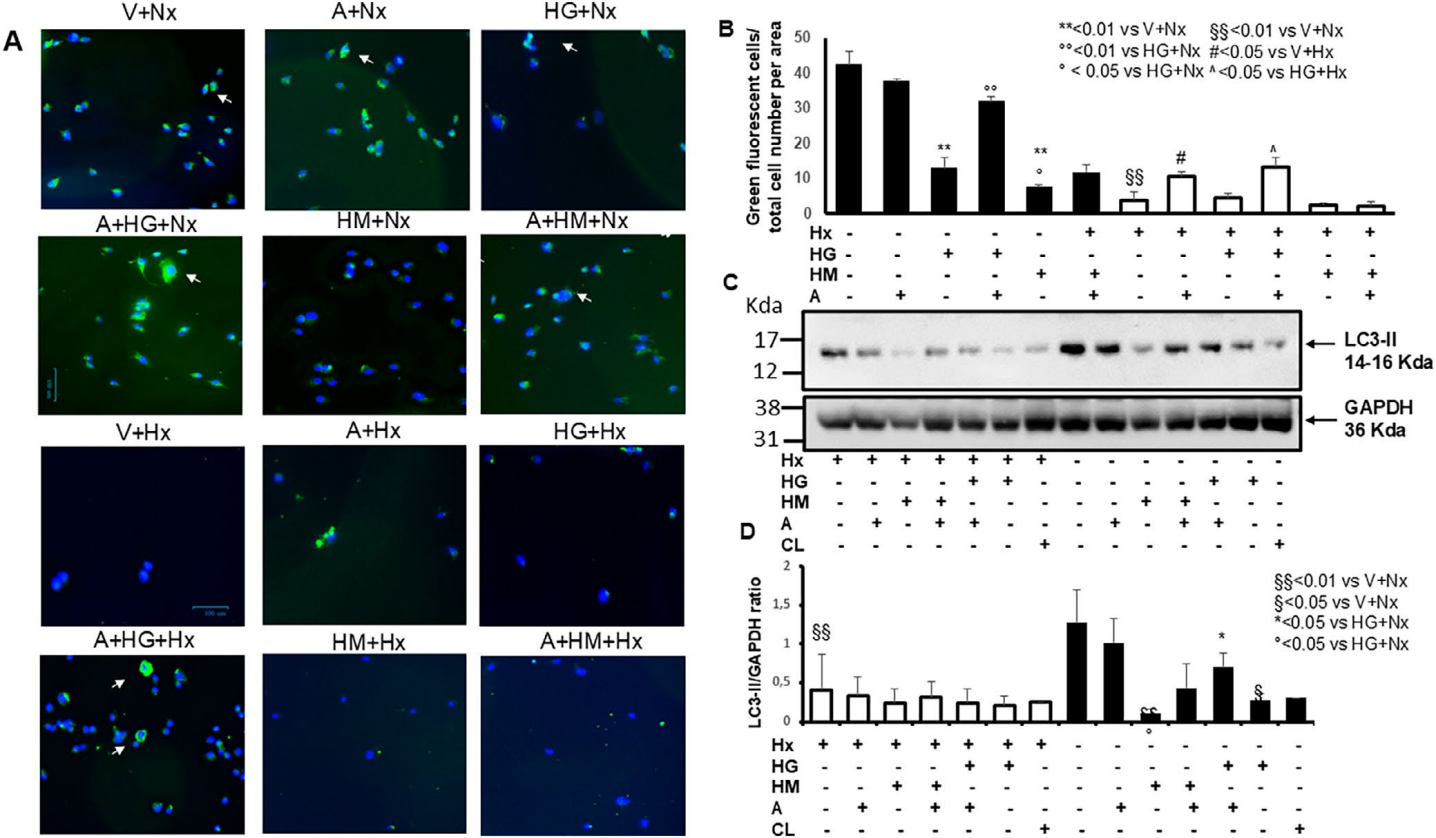
Ambrisentan increased autophagy in hPAECs treated with high glucose under hypoxia/normoxia. (A) Representative immunofluorescence microscopy images of green detection reagent indicating the presence of autophagic vacuoles in hPAECs treated for 24 h with control D‐glucose (V), high glucose (HG) or high mannitol (HM), in the presence or absence of 0.02 nM ambrisentan, under normoxia (A+Nx) and hypoxia (A+Hx). Scale bars 100 μm. Original magnification ×200 and ×400. (B) The graph represents image analysis of autophagic vacuole immunofluorescence percentage reported as mean ± SD. Each experiment was repeated three times, analysing every time at least 8 microscopic fields per group. Statistical analysis was performed by one‐way ANOVA followed by Tukey's multiple comparison. **p < 0.01 vs. V+Hx, °*p* < 0.05 and °°*p* < 0.01 vs. HG+Nx, §§*p* < 0.01 vs. V+Nx, #*p* < 0.05 vs. V+Hx, ^*p* < 0.05 vs. HG+Hx. (C) Representative western blot of autophagic marker LC3‐II on hPAECs treated for 24 h with high glucose (HG) or high mannitol (HM), in the presence or absence of 0.02 nM ambrisentan, under normoxia (A+Nx) and hypoxia (A+Hx). In parallel experiments, cells were treated with chloroquine (CL) as a positive control for autophagy. GAPDH was used as internal control. In this representative western blot, the same membrane was first incubated with anti‐LC3‐II, then stripped and reincubated with anti‐GAPDH. (D) In the graph, results are reported as LC3‐II/GAPDH ratio of the optical density of matching bands and are mean ± SD of triplicate experiments. Statistical analysis was done by one‐way ANOVA with Tukey test for multiple comparisons, *p < 0.05 vs. V+H; §*p* < 0.05 and §§*p* < 0.01 vs. V+*N*; °*p* < 0.05 vs. HG + *N*.

Autophagy plays an important role in the development of PH, and targeting the formation and maturation of autophagosomes has recently emerged as a new modality for treating pulmonary hypertension [21]. Autophagy upon ambrisentan and HG or HM in hPAECs exposed to normoxia/hypoxia was explored by monitoring the presence of fluorescent autophagosomal vacuoles, by microscopic analysis, and quantified by immunofluorescence intensity. Under normoxic conditions, HG induced a smaller decrease in autophagy when compared to HM, which strongly reduced autophagy. We observed a significant reduction in autophagosome accumulation in hPAECs under Hx alone compared to normoxia, by at least 8‐fold. AMB alone increased autophagosome accumulation in hPAECs under Hx but not Nx, by about twofold compared to control or vehicle (Figure 2A,B). Coincubation with ambrisentan and HG reversed the HG‐induced autophagosome decrease (Figure 2A,B), either in Nx or in Hx.

In the western blot analysis, LC3‐II expression was strongly inhibited by Hx compared to Nx (Figure 2C,D). Similarly, HG and HM reduced LC3‐II expression in Nx compared to vehicle (Figure 2C,D), but only HG was reversed by AMB.

Taken together, autophagy in hPAECs is inhibited by Hx and HG/HM, and clearly induced by AMB.

### 3.3 Ambrisentan Up‐Regulates the Expression of miR124‐3p in hPAECs Treated With High Glucose Under Normoxic Conditions and High Mannitol Under Normoxic and Hypoxic Conditions

**FIGURE 3:**
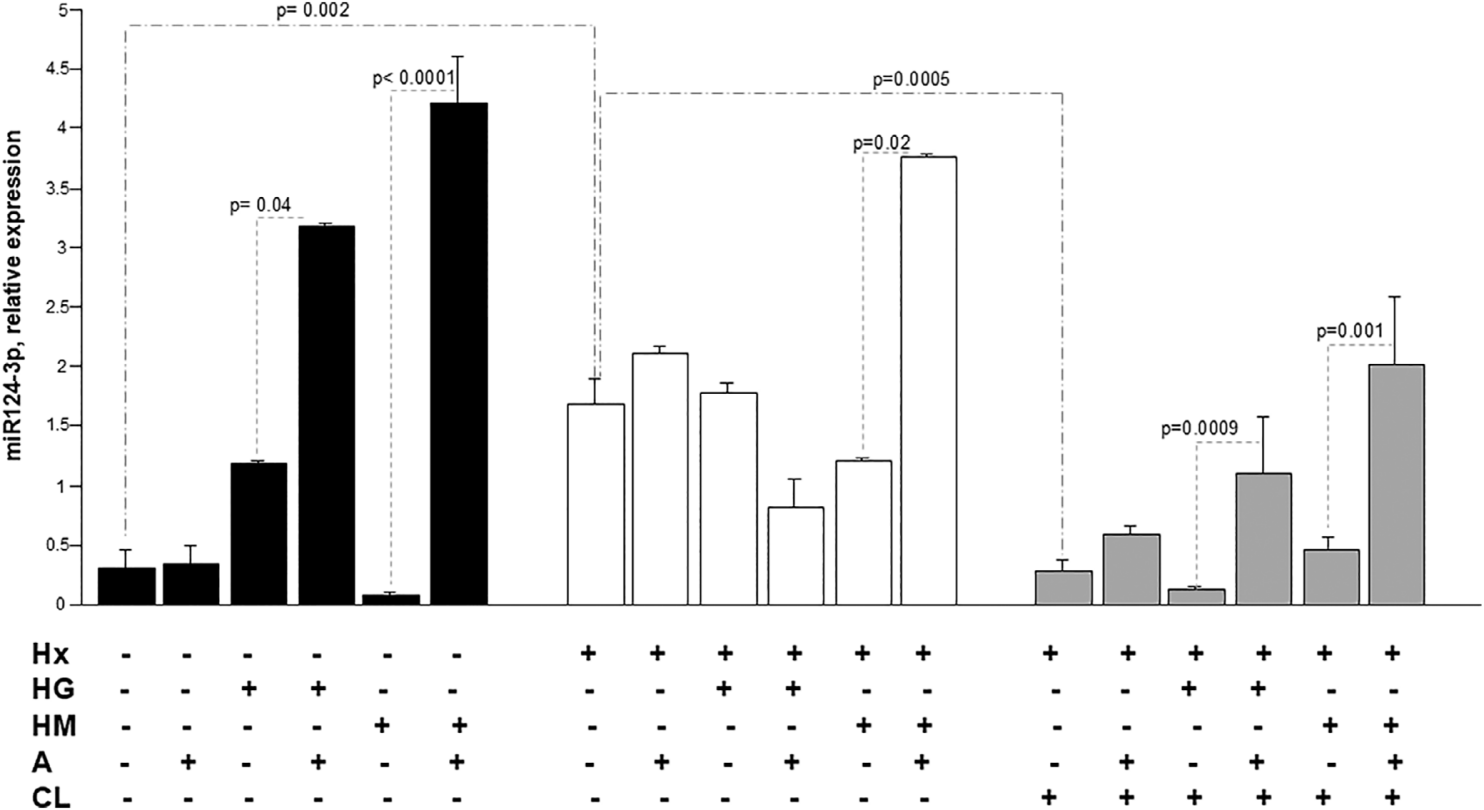
Ambrisentan up‐regulates the expression of miR124‐3p in hPAECs treated with high glucose and high mannitol under normoxia or with high mannitol under hypoxia. miR124‐3p expression in hPAECs incubated with control d‐glucose concentration (Vehicle, V), high glucose (HG), high mannitol (HM), for 24 with/without 0.02 nM ambrisentan (AMB) in normoxia (Nx, black bars) or hypoxia (Hx, white bars). miR124‐3p expression in HPAECs incubated with control d‐glucose concentration (Vehicle, V), high glucose (HG), high mannitol (HM), for 24 with/without 0.02 nM ambrisentan (AMB) in hypoxia (H, grey bars) in the presence of chloroquine (CL). Results are reported as relative expression of miR124‐3p are mean ± SEM of triplicate experiments. Statistical analysis was done by one‐way ANOVA with Fisher's test for multiple comparisons; comparisons among groups and corresponding *p* values were reported on the graphs.

**FIGURE 4:**
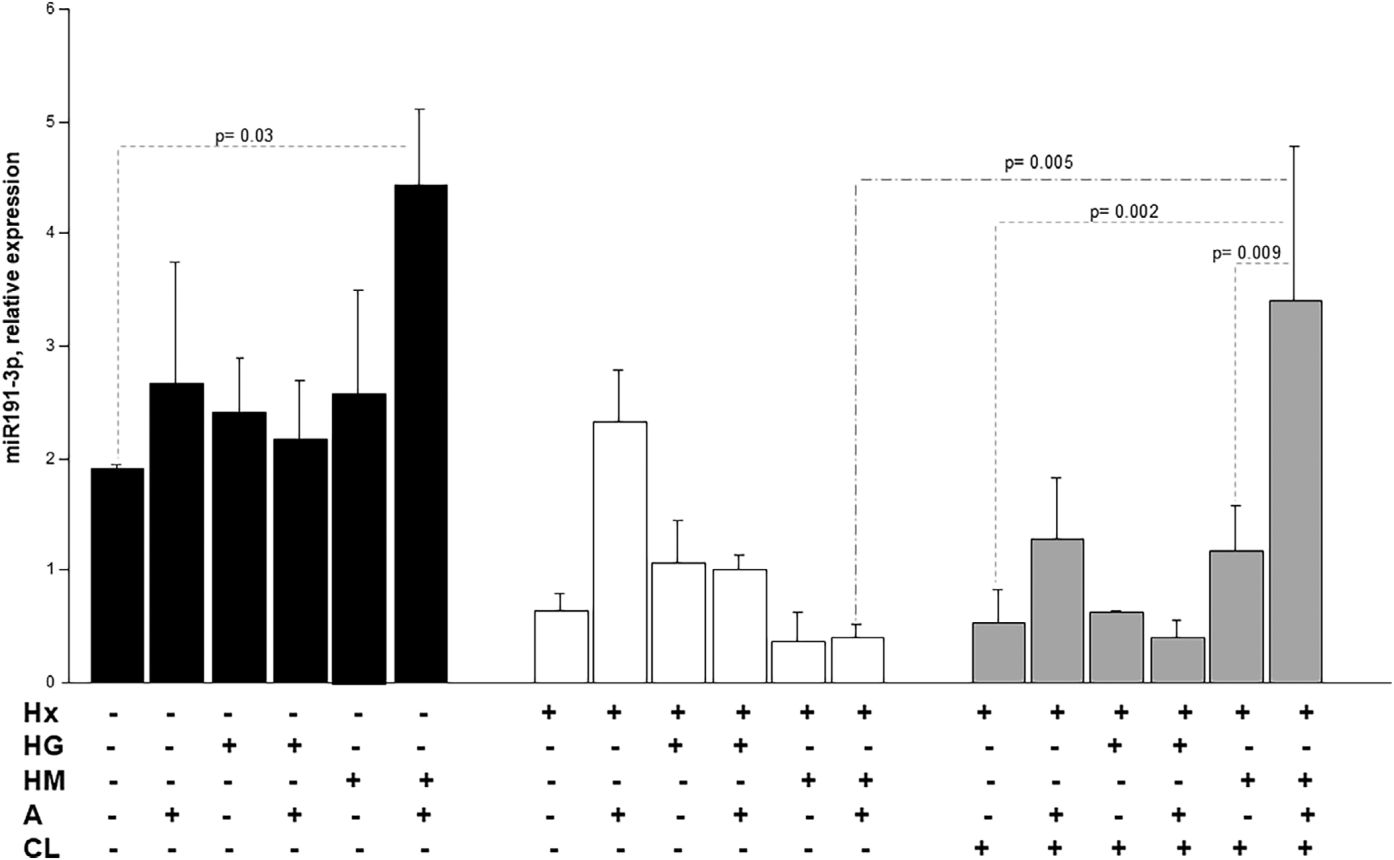
Ambrisentan up‐regulates the expression of miR191p in hPAECs treated with high mannitol under normoxia. miR191‐3p expression in hPAECs incubated with control d‐glucose concentration (Vehicle, V), high glucose (HG), high mannitol (HM), for 24 with/without 0.02 nM ambrisentan in normoxia (A+*N*, black bars) or hypoxia (A+H, white bars). miR191‐3p expression in HPAECs incubated with control d‐glucose concentration (Vehicle, V), high glucose (HG), high mannitol (HM), for 24 with/without 0.02 nM ambrisentan in hypoxia (A+H, grey bars) in the presence of chloroquine (CL). Results are reported as relative expression of miR191‐3p and are mean ± SEM of triplicate experiments. Statistical analysis was done by one‐way ANOVA with Fisher's test for multiple comparisons; comparisons among groups and corresponding *p* values were reported on the graphs.

We assessed by real‐time PCR the expression of miR124‐3p and miR‐1919‐3p, which play a key role in pulmonary arterial remodelling [14]. As shown in Figures 3 and 4, both miR‐124‐3p and miR‐191‐3p are expressed in hPAEC, both under Nx and Hx and in all treatment conditions. Specifically, compared to Nx in vehicle (V)‐treated hPAEC, Hx increased the abundance of antiapoptotic miR124‐3p (*p* = 0.002) (Figure 3B), and induced an opposite effect on antiapoptotic and proliferative miR191‐3p (Figure 4B), although not statistically significant.

In Nx, AMB potentiated the expression of miR124‐3p in HG‐treated hPAEC (*p* = 0.04) and in HM‐treated hPAEC (*p* < 0.0001) (Figure 3A) but not miR191‐3p (Figure 4A), and only miR191‐3p in HM‐treated hPAEC compared to vehicle (*p* = 0.03) (Figure 4A). In Hx, only AMB increased miR‐124‐3p in HM‐treated hPAEC (*p* = 0.02) (Figure 3B), while no treatment modulated miRNA‐191‐3p expression to a statistically significant extent (Figure 4B). Conversely, HG treatment with or without AMB had no impact on the expression of miR124‐3p nor miR‐191‐3p in Hx.

### 3.4 Autophagy Blockade Restores Ambrisentan‐Induced miR‐124‐3p Upregulation in hPAECs Treated With High Glucose Under Hypoxic Conditions and Promotes Ambrisentan‐Induced miR‐191‐3p Upregulation in hPAECs Treated With High Mannitol Under Hypoxic Conditions

To examine the existence of any relationship between autophagy and miR‐191‐3p or miR‐124‐3p and see whether the effects of Hx and AMB on the two miRNAs are mediated by autophagy, their expression was investigated under the blockade of autophagy by chloroquine (CL). In Hx, chloroquine significantly reduced the expression of miR‐124‐3p in the CL_H group compared to the V_Hx group, and in cells treated with HG and HM, suggesting an intimate mechanistic link between autophagy and miR‐124‐3p modulation by hypoxia (*p* = 0.0005, Figure 3C). However, under Hx, autophagy blockade did not reverse the effect of AMB on miR124‐3p expression in hPAECs treated with HM. Instead, it restored the miR124‐3p‐inducing effect of AMB in hPAECs treated with HG (Figure 3C) and the miR191‐3p‐inducing effect of AMB in hPAECs treated with HM (Figure 4C). These data suggest that autophagy is involved in the regulatory effect of ambrisentan on miRNA expression under hypoxia, differently depending on the type of miRNA and the co‐morbidity mimicked in vitro. Specifically, autophagy mediates the effect of AMB on miR124‐3p in hPAECs exposed to Hx and HG and on miR191‐3p in hPAECs exposed to Hx and HM.

No significant modulation of miR146a‐3p, miR7110‐3p and miR193b‐3p was observed by AMB in either of the experimental co‐morbidities tested or oxygen condition (data not shown).

### 3.5 Ambrisentan Down‐Regulates Apoptosis in hPAECs Treated With High Glucose Under Normoxic Conditions and High Mannitol Under Normoxic and Hypoxic Conditions

**FIGURE 5:**
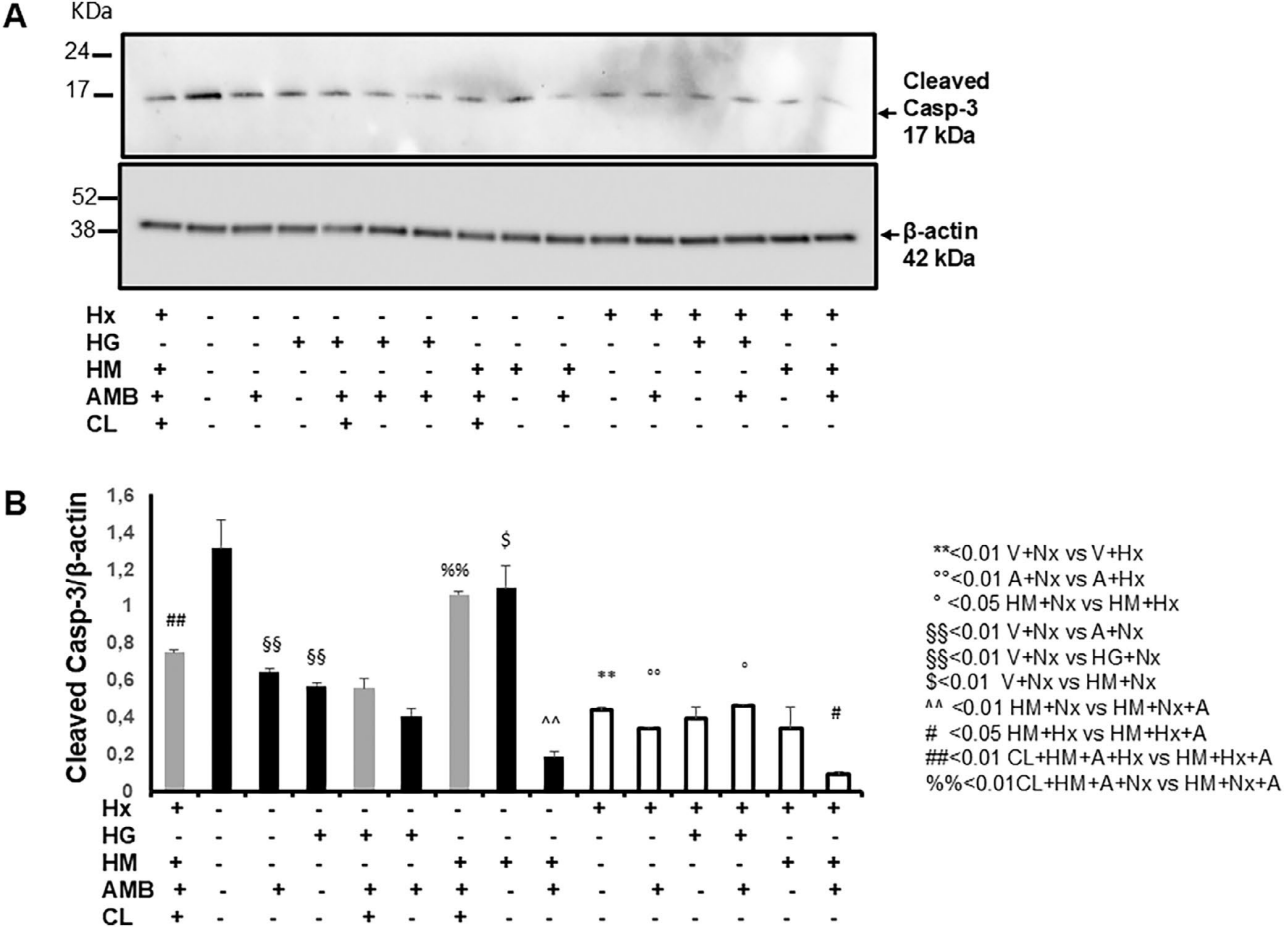
Ambrisentan bluntes apoptosis in hPAECs treated with high glucose under normoxic conditions and high mannitol under normoxic and hypoxic conditions. (A) Representative western blot of cleaved caspase‐3 in hPAECs treated for 24 h with high glucose (HG) or high mannitol (HM), in the presence or absence of 0.02 nM ambrisentan (AMB), under normoxia (A+Nx) and hypoxia (A+Hx). In parallel experiments, cells were treated with chloroquine (CL). β‐Actin was used as internal control. In this representative western blot, the same membrane was first incubated with anti‐cleaved caspase 3, then stripped and reincubated with anti‐ β‐Actin. (B) In the graph, results are reported as cleaved caspase 3/β‐Actin ratio of the optical density of matching bands and are mean ± SD of triplicate experiments. Statistical analysis was done by one‐way ANOVA with Tukey test for multiple comparisons, *p < 0.05 vs. V+H; §*p* < 0.05 and §§*p* < 0.01 vs. V+*N*; °*p* < 0.05 vs. HG+*N*. Statistical analysis was performed by one‐way ANOVA followed by Tukey's multiple comparison: ** < 0.01 V+Nx vs. V+Hx; °° < 0.01 AMB+Nx vs. A+Hx; *< 0.05 HG+Nx vs. HG+Hx; ° < 0.05 HM+Nx vs. HM+Hx; ^ < 0.05 HM+Nx+AMB vs. HM+Hx+A; §§ < 0.01 V+Nx vs. AMB+Nx; §§< 0.01 V+Nx vs. HG+Nx; $$ < 0.01 V+Nx vs. HM+Nx; § < 0.05 HG+Nx vs. HG+Nx+AMB; ^^ < 0.01 HM+Nx vs. HM+Nx+AMB; # < 0.05 HM+Nx vs. HM+Nx+AMB; ## < 0.01 CL+HM+AMB+Hx vs. HM+Hx+AMB; ++< 0.01 CL+HG+AMB+Nx vs. HG+Nx+AMB; %% < 0.01 CL+HM+AMB+Nx vs. HM+Nx+AMB.

To validate miRNA expression data, the expression of cleaved caspase‐3 was investigated under the blockade of autophagy by chloroquine (CL). As shown in Figure 5A,B, hypoxia induced an overall reduction in cleaved caspase‐3, compared to control (V+Nx), despite causing an increase in LDH release (Figure 1C), suggesting that oxygen deprivation induces cell death by necrosis but spares cell death from apoptosis. Interestingly, the process was exacerbated by co‐incubation of AMB and HM, with a reduction in the optical density of cleaved caspase‐3 of at least 4‐fold compared to V+Hx (Figure 5B), despite not triggering cell death by necrosis (Figure 1C). Similar results were observed in Nx, where treatments with AMB decreased apoptosis, either alone or in the presence of HM. Chloroquine significantly restored apoptosis blunted by AMB in HM, suggesting that autophagy is involved in the regulatory effect of ambrisentan on apoptosis, in line with observations on miR124‐3p.

## 4 Discussion

In this study, we demonstrated that: (1) AMB had a cytotoxic effect in Nx in a concentration‐dependent manner; (2) in Hx, at non‐cytotoxic concentrations ranging from 0.2 to 0.02 nM, AMB was unable to prevent cytotoxicity either in the presence or absence of HG, whereas HM did not exert any effect; (3) high glucose exerted an anti‐autophagic effect partly through a hyperosmolar stress‐related mechanism only in Nx; (4) AMB was associated with increased autophagic activity (autophagosome formation) in the presence of high glucose and, to a lesser extent, in the presence of high mannitol, both in Nx and Hx. This suggests that AMB totally counteracts the anti‐autophagic effect of high glucose, likely due to its metabolic component, and partly related to its hyperosmolar component. Lastly, AMB‐treated hPAECs displayed higher miR‐124‐3p expression under high glucose and high mannitol conditions in Nx, but only under high mannitol conditions in Hx. Hypoxia is a condition that evokes deep changes in cell metabolism. Above all, the use of glucose as exclusive fuel since other energy sources are metabolised only in the presence of oxygen, which is a strong inhibitor of glucose metabolism [25]. The consumption of glucose might have blunted the hyperosmolar stress induced by its high levels.

Moreover, the Hx‐inducing effect of miRNA‐124‐3p appears to be mediated by autophagy, as it is reversed by chloroquine, therefore AMB seems to act on miRNA‐124‐3p in an autophagy‐dependent manner.

In the absence of clear data available in the literature on the exact equivalence of in vitro concentrations with the doses of the drug used in patients, we generated our own cytotoxicity curve, and we found that the drug exerted cytotoxicity on hPAEC starting at 0.02 nM even in Nx and in the absence of HG or HM. A paper [12] reported that the pKB (the antagonist dissociation constant) estimates obtained for drug concentrations between 0.1 and 1 μM would give a significant block of the 22‐fold shift in the endothelin‐1 (ET1) concentration‐response curves of the radial artery and a 55‐fold shift in the pulmonary artery. Thus, the in vitro concentrations tested in our study fall well within the drug doses effective in vivo.

The wealth of data collected in our experiments needs to be put into perspective with existing evidence, so as to grasp its full implications for the issue of comorbid PAH. A better understanding of the effect of treatment on hPAEC viability, autophagy, and transcriptome across different and varying external conditions could have important translational implications.

### 4.1 The Effect of Ambrisentan on hPAEC Viability

Our experiments showed that AMB exerts a cytotoxic effect on hPAECs at a concentration < 0.02 nM, with a two‐step dose–response curve (Figure 1A). To our knowledge, this effect has never been documented before. Still, endothelin 1 (ET1), a well‐known mediator of pulmonary vascular remodelling, has been linked to endothelial cell apoptosis and smooth muscle cell survival [13]. As such, endothelial cells in PAH group1, PAH group 4 and PAH group 3 express increased levels of ET1. If this is the case, given the antiremodeling effect of ERAs, the pro‐apoptotic effect of AMB is apparently contradictory. However, a pro‐proliferative action is equally unlikely, since a study contrasting bosentan to sildenafil found that the ERAs do not induce angiogenesis either in vitro or in vivo [13]. The question arises whether AMB may induce apoptosis in endothelial cells selectively, possibly depending on their phenotype, and thus counter pulmonary vascular remodelling. This theory would resonate with the non‐linearity of the dose–response curve: the cytotoxic effect starts at 10 nM and reaches a plateau, which could mean that all apoptosis‐susceptible endothelial cells have been eliminated. Importantly, AMB‐mediated cytotoxicity was not replicated in the second phase of the viability assessment (Figure 1B), regardless of Hx and co‐treatment with HG or HM. These results were confirmed also by the LDH activity assay (Figure 1C). The fact that Hx resulted in a reduction in cell survival across all culture media seems to confirm the validity of these findings. Interestingly, in our hPAEC model, AMB blunted apoptosis in Nx conditions in the presence or absence of HM, even though it failed to exert an appreciable detrimental effect on cell viability or cell necrosis.

### 4.2 The Effect of Ambrisentan and Hypoxia on Autophagy in hPAECs

The role of autophagy in PAH in particular has been widely investigated, but it still remains unclear whether it mediates or protects against pulmonary vascular disease. A number of substances and drugs have been found to exert favourable effects on PAH by inhibiting autophagy [26, 27, 28, 29, 30, 31] and an increased autophagic flux has been linked to pulmonary vascular disease [13, 32, 33]. On the other hand, however, a protective role of the autophagic protein LC3B has also been reported [34]. An interesting review on the topic has synthesised existing evidence on the issue and has referred to autophagy as a double‐edged sword in PAH [35]. Most studies on autophagy in PAH involved experimental models, including monocrotaline‐induced PAH and hypoxic PAH [31, 33]. Hx activates autophagy in pulmonary artery smooth muscle cells [28, 31], while its effect on hPAECs is less clear. Zhang et al. [13] have recently concluded that Hx upregulates the autophagic flux in pulmonary artery endothelial cells regardless of their phenotype (hPAECs vs. hMVECs), suggesting that the effect of hypoxia on autophagy is not limited to the pulmonary macrovasculature but also extends to the microvasculature.

On the contrary, Hx negatively modulated autophagy in our experiment: compared to Nx, Hx was associated with a reduction of autophagy across all culture media (Figure 2A,B). AMB was associated with an increased autophagic activity in terms of autophagosome formation in HG regardless of oxygen tension. This effect, which was not observed in normoxic cells treated with a vehicle and in the hyperosmolar, could be one of the cellular correlations of the reduced efficacy of ERAs in the comorbid setting of diabetes. In terms of LC3 expression, the effect of AMB on HG and HM was comparable in Hx conditions. It is noteworthy that autophagosome formation more comprehensively probes autophagy, while LC3 is only one of the autophagy markers. To our knowledge, the effect of ET1 inhibition on autophagy has not been systematically addressed, but ET1 was shown to mediate NETosis (a specific form of neutrophil cell death) through the upregulation of autophagy in a study conducted on neutrophils in systemic lupus erythematosus [36]. Further studies on the topic are needed to confirm this hypothesis, namely that ERAs may have a neutral or inhibitory effect on autophagy in hPAECs but turn into an autophagy upregulator in the presence of a specific comorbid context (Figure 2B).

### 4.3 Ambrisentan, miR‐124‐3p and miR‐191‐3p

Growing evidence has been collected on the involvement of non‐coding RNAs in a number of diseases [37], including PAH [38, 39]. Several miRNAs have been associated with PAH and, despite a certain lack of concordance between animal and human studies [13], some of these have been suggested and investigated as useful disease markers and/or therapeutic targets [40]. miR‐124, which was reported to mediate proliferation and inflammation [40, 41, 42], is downregulated in the lungs of PAH patients [13]. Its downregulation in PAH hPAECs has been associated with the dysregulation of the NOTCH1 and PTEN pathways [42], to cell proliferation and migration [43], and to the Warburg‐like metabolic shift from mitochondrial respiration to glycolysis [44]. All of these are central features of PAH pathogenesis. miR‐191 has also been implicated in specific abnormalities in pulmonary vascular disease. Its levels were elevated in RV pressure overload due to PAH compared to matching controls [45]. This suggests that it might be directly implicated in PAH, yet its increase in a rat model of PAH inhibited the expression of bone morphogenic protein receptor 2 in hPAECs and seemed thus protective. Hx has been shown to regulate the expression of a number of miRNAs. These are collectively referred to as hypoxamirs [46]. It was reported that miR‐191 increased under hypoxic conditions [47], while miR‐124 is apparently downregulated in pulmonary artery smooth muscle cells exposed to chronic Hx [41]. In our study, only miR124‐3p was upregulated by Hx in hPAECs exposed to high glucose, in an autophagy‐dependent manner. The highest level of expression of miR‐124 was found in hPAECs incubated with AMB in a hyperglycemic medium, under Nx and in a hyperosmolar medium both under Nx and Hx (Figure 3A,B), in an autophagy‐independent manner. On the contrary, AMB was able to modulate miR‐191 expression in a hyperosmolar medium under Nx. This effect is restored in hypoxia only after blocking autophagy. A favourable action on hPAEC transcriptome under hyperglycemic and/or hypoxic conditions may represent one of the mechanisms driving the beneficial action of ERA inhibition in the comorbid setting. No data exist on the specific effect of ERAs and other PAH treatments on the cell transcriptome, even though all three pathophysiological axes of pulmonary vasodilation have been linked to miRNA expression [36, 48, 49]. Further studies, possibly involving animal models, are warranted to confirm and understand the implications of ERAs‐mediated rise in miR‐124 and miR‐191.

### 4.4 Limitations

Several limitations apply to our study. This was not conducted on hPAECs from PAH patients with/without comorbidities, which may undermine the validity of our findings in the real PAH context. Furthermore, AMB findings should be validated by other ERAs. Secondly, the exploratory assumption that specific in vitro conditions may reproduce the effects of a real‐world comorbid setting is simplistic, and the results should be regarded as thought‐provoking hypotheses that deserve to be tested in specific organoids or animal models of PAH. Finally, as already detailed, the cytotoxicity assay using absorbance as a parameter failed to show a predictable effect of hyperosmolarity and may have suffered from a lack of sensitivity.

### 4.5 Clinical Perspective and Conclusions

The effect of targeted therapies on PAH is heterogenous, but the relevance of comorbidities on patient treatment response is not clear. Pulmonary hypertension (PH) associated with left heart disease can coexist with PAH. These patients, who are affected by a peculiar form of combined pre‐ and post‐capillary PH, are incorrectly comprised in the group of PAH patients with cardiac comorbidities. These represent a significant portion of misclassified PAH and respond suboptimally to the pulmonary vasodilatory treatment both in terms of efficacy and tolerability. Our hypothesis, evaluated here, is that comorbidities can give a suboptimal response to PAH‐specific therapies regardless of misclassification. They can interfere with the response of pulmonary endothelial cells to PAH‐specific drugs, making them resistant to their vasodilatory and antiremodeling action.

In hPAEC exposed to Hx, AMB retains its pro‐autophagic effects in an in vitro model mimicking diabetes. In Nx, AMB potentiated the expression of the antiapoptotic miR124‐3p in HG‐treated hPAEC, and only in HM‐treated hPAEC under Hx. miR124‐3p and, to a lesser extent, miR191‐3p, may act as biomarkers of disease and treatment response to specific drugs in patients with PAH. Such findings warrant future studies that could further identify the mechanisms by which pulmonary endothelial cells convey instructive and inductive signals that impair PAH response to treatment in the presence of comorbidities.

## Author Contributions

**Manuela Cabiati:** data curation (equal), investigation (equal), methodology (equal), writing – original draft (equal). **Filippo Biondi:** conceptualization (equal), investigation (equal), writing – original draft (equal), writing – review and editing (equal). **Sandra Ghelardoni:** investigation (equal), project administration (equal), writing – original draft (equal). **Valentina Casieri:** formal analysis (equal), investigation (equal), methodology (equal). **Vincenzo Lionetti:** formal analysis (equal), funding acquisition (equal), project administration (equal), writing – original draft (equal). **Agnese Sgalippa:** data curation (equal), investigation (equal), methodology (equal). **Silvia Del Ry:** investigation (equal), methodology (equal), supervision (equal), supervision (equal), writing – original draft (equal), writing – original draft (equal). **Rosalinda Madonna:** conceptualization (equal), data curation (equal), formal analysis (equal), funding acquisition (equal), investigation (equal), methodology (equal), project administration (equal), resources (equal), software (equal), supervision (equal), validation (equal), writing – original draft (equal), writing – review and editing (equal).

## Conflicts of Interest

The authors declare no conflicts of interest.

## Supporting information


**Figure S1.**Representative images of human pulmonary artery endothelial cells (HPAC), observed in Nomarski interference contrast, incubated with control d‐glucose concentration (Vehicle), high glucose (HG), high mannitol (HM), for 24 with/without 0.02 nM ambrisentan (A) in normoxia.

**Figure S2.**Representative images of human pulmonary artery endothelial cells (HPAC), observed in Nomarski interference contrast, incubated with control d‐glucose concentration (Vehicle), high glucose (HG), high mannitol (HM), for 24 with/without 0.02 nM ambrisentan (A) in hypoxia.

Data S1.

Data S2.

Data S3.

## Data Availability

Data available on request from the authors.
