# Supplementary figures and images for: Ambrisentan Retains Its Pro‐Autophagic Activity on Human Pulmonary Artery Endothelial Cells Exposed to Hypoxia in an In Vitro Model Mimicking Diabetes
Source: J Cell Mol Med. 2025 Apr 9;29(7):e70528. doi: 10.1111/jcmm.70528 (PMC11982177; doi:10.1111/jcmm.70528)

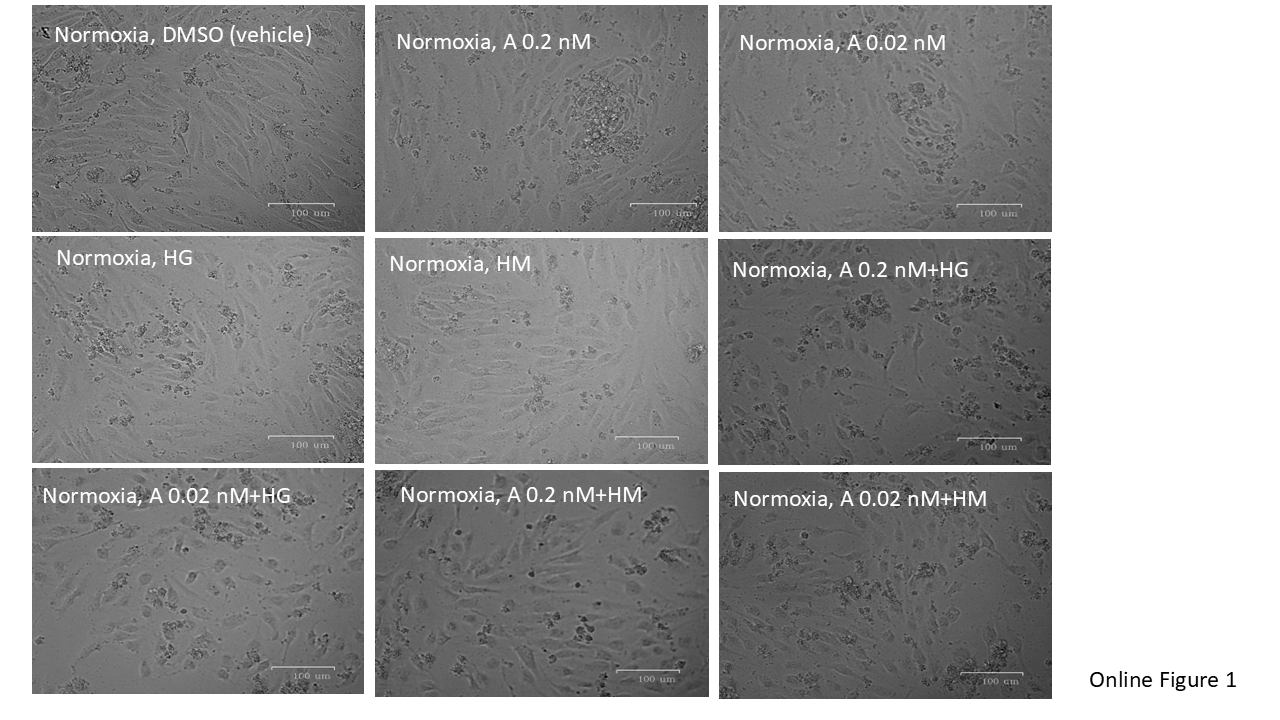

Supplement: Supplementary file 1 — Figure S1.Representative images of human pulmonary artery endothelial cells (HPAC), observed in Nomarski interference contrast, incubated with control d‐glucose concentration (Vehicle), high glucose (HG), high mannitol (HM), for 24 with/without 0.02 nM ambrisentan (A) in normoxia. [file JCMM-29-e70528-s004.tif]

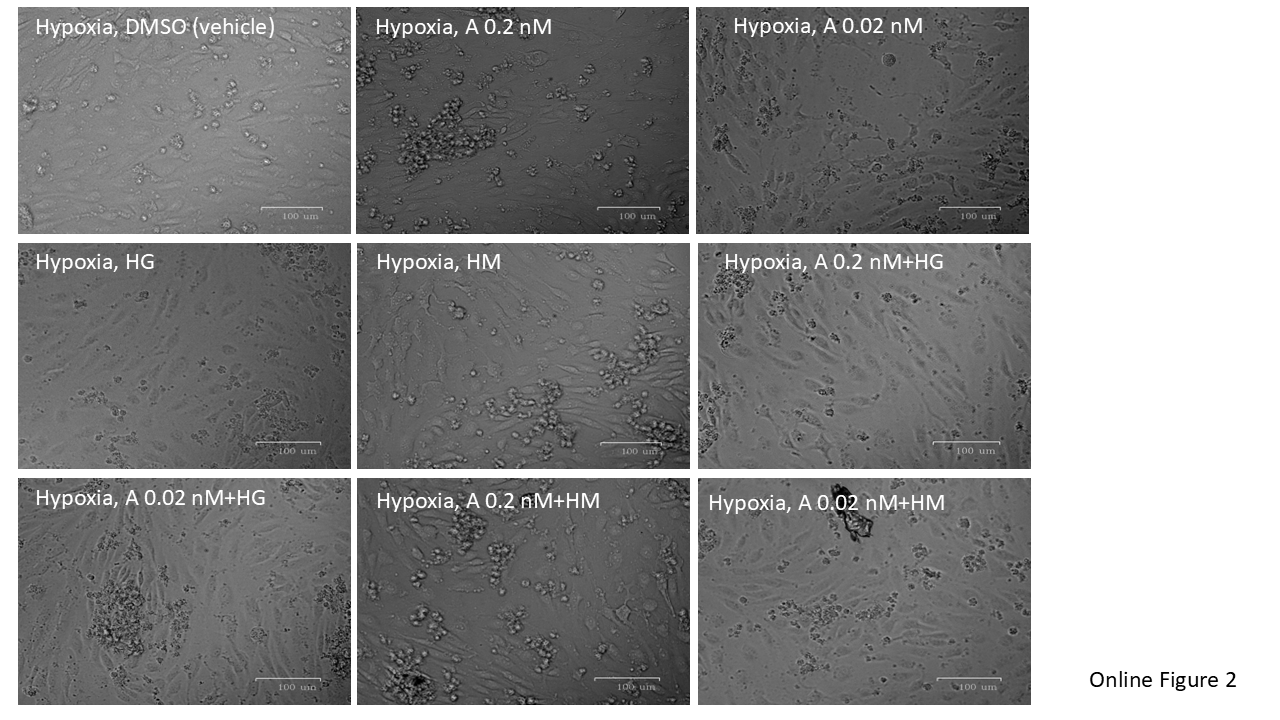

Supplement: Supplementary file 2 — Figure S2.Representative images of human pulmonary artery endothelial cells (HPAC), observed in Nomarski interference contrast, incubated with control d‐glucose concentration (Vehicle), high glucose (HG), high mannitol (HM), for 24 with/without 0.02 nM ambrisentan (A) in hypoxia. [file JCMM-29-e70528-s005.tif]
