# Supplementary material for: Ambrisentan Retains Its Pro‐Autophagic Activity on Human Pulmonary Artery Endothelial Cells Exposed to Hypoxia in an In Vitro Model Mimicking Diabetes
Source: J Cell Mol Med. 2025 Apr 9;29(7):e70528. doi: 10.1111/jcmm.70528 (PMC11982177; doi:10.1111/jcmm.70528)
Supplement: Supplementary file 3 — Data S1. [file JCMM-29-e70528-s002.docx]

| **GENE** | **FORWARD PRIMER SEQUENCE**  **(5’---3’)** | **GENBANK,**  **ACCESSION NUMBER** | **LOCATION** | **Ta, °C** |
| --- | --- | --- | --- | --- |
| **hsa-miR-124-3p** | TAAGGCACGCGGTGAATGCCAA | NR029670.1 | chr 20q13.33 | 55 |
| **hsa-miR-191-3p** | GCTGCGCTTGGATTTCGTCCCC | NR029690.1 | chr 3p21.31 | 55 |
| **hsa-miR-193b-3p** | AACTGGCCCTCAAAGTCCCGCT | NR_030177 | chr 16p13.12 | 55 |
| **hsa-miR-146a-3p** | CCTCTGAAATTCAGTTCTTCAG | NR_029701 | chr 5q33.3 | 55 |
| **hsa-miR-7110-3p** | TCTCTCTCCCACTTCCCTGCAG | NR_106960 | chr 3q21.1 | 55 |

**Online Table 1:** **Mature miRNA sequence**

**Table legend.** **hsa-miR-124-3p:** homo sapiens microRNA-124 with 3p strand present in the reverse position; **hsa-miR-191-3p:** homo sapiens microRNA-191 3p strand present in the reverse position; **hsa-miR-193b-3p:** homo sapiens microRNA-193b 3p strand present in the reverse position; **hsa-miR-146a-3p:** homo sapiens microRNA-146a 3p strand present in the reverse position; **hsa-miR-7110-3p:** homo sapiens microRNA-7110 3p strand present in the reverse position.
