# Supplementary material for: Ambrisentan Retains Its Pro‐Autophagic Activity on Human Pulmonary Artery Endothelial Cells Exposed to Hypoxia in an In Vitro Model Mimicking Diabetes
Source: J Cell Mol Med. 2025 Apr 9;29(7):e70528. doi: 10.1111/jcmm.70528 (PMC11982177; doi:10.1111/jcmm.70528)
Supplement: Supplementary file 4 — Data S2. [file JCMM-29-e70528-s001.docx]

**Ambrisentan retains its pro-autophagic activity on human pulmonary artery endothelial cells exposed to hypoxia in an *in vitro* model mimicking diabetes**

Manuela Cabiati^1§^, Filippo Biondi^2§^, Sandra Ghelardoni^3^, Valentina Casieri^4^, Agnese Sgalippa^1^, Silvia Del Ry^1, 4^, Rosalinda Madonna^1,2*^

^§^ equally contributed

^1^ Laboratory of Biochemistry and Molecular Biology, Institute of Clinical Physiology, CNR, 56124 Pisa, Italy

^2^ Department of Pathology, Cardiology Division, University of Pisa, Pisa, Italy

^3^ Department of Pathology, Laboratory of Biochemistry, University of Pisa, Italy

^4^ Unit of Translational Critical Care Medicine, Laboratory of Basic and Applied Medical Sciences, Interdisciplinary Research Center "Health Science," Scuola Superiore Sant'Anna, Pisa, Italy

**Online Supplement**

**MATERIALS AND METHODS**

Ambrisentan, D-glucose and D-mannitol were purchased from Sigma Aldrich (St Louis, Missouri). Human pulmonary artery endothelial cells (hPAECs) were purchased from Cascade Biologicals (Portland, Oregon).

**Cell cultures and treatments**

HPAECs (Cat. no C-008-5C, Lot#6C0189, Cascades Biologicals, USA) were maintained in Medium 231 with low serum growth supplement (Life technologies, USA). The cells were used in the study from passage 3 to 5. Hypoxia was induced by exposing the cells to 4% O_2_ with 4% CO_2_ and 92% N_2_ for 24 h at 37°C. Cells were incubated with control d-glucose concentration (Vehicle, 5.5 mmol/L, control and 285 mOsm/L), high glucose (HG: 30.5 mmol/L and 385 mOsm/L), high mannitol (HM: 5.5 mmol/L glucose + 25 mmol/L and 385 mOsm/L), for 24 with/without ambrisentan (0.02 nM) in normoxia (N) or hypoxia (H). At the end of treatments cells were harvested for RNA isolation, or incubated with specific dyes for MTT and autophagy assays.

**Measurement of cell viability**

The cytotoxic effect of high glucose and high mannitol with or without ambrisentan was determined using a 3-(4,5-Dimethylthiazol-2-yl)-2,5-diphenyltetrazolium bromide assay (MTT, Sigma). hPAECs were harvested, diluted to 2 x 10^4^ cell per 100 µL, and seeded in 96 well plates. hPAECs were treated with HG, HM or vehicle (Con, DMSO) or ambrisentan (0.02 or 0.2 nM), in single or cotreatment, under hypoxia or normoxia conditions for 48 hours. To assess ambrisentan’s toxicity, hPAECs were treated with ambrisentan in a concentration range from 0.02 to 5000 nM, for 48h. At the end of incubation, MTT test was performed. Briefly, MTT (0.5 mg/ml) was added to the medium, and after 4 h an SDS–HCl solution (0.05 mg/ml) was used to solubilize the formed formazan salt. The absorbance of the solution was read at 570 nm after 18h in a microplate reader (BioRad Laboratories, Italy). Results were expressed as percentage of control for ambrisentan toxicity or as absorbance at 570 nm in the overall evaluation. All samples were tested in 8 replicates

**Autophagy detection by immunofluorescence**

The effect of HG, HM with or without ambrisentan in hypoxia and normoxia conditions on autophagy of live hPAECs was detected by Autophagy Detection Kit (Abcam ab139484, Cambridge, UK) accordingly with vendor’s protocol (*n* = 3 indipendent experiments). hPAECs were plated at 2 × 10^5^ cells per well in a 6-well chamber slides, grown overnight, then exposed to normoxia or hypoxia and treated with HG, HM, or ambrisentan in single or cotreatment for 24 hours. hPAECs were treated as follow. After blocking with 1% BSA (Sigma-Aldrich) for 30 min at room temperature, fluorescent dyes for nuclei staining and autophagy detection were added and incubated for 30 min at room temperature. After three washing using PBS, the autophagic vacuoles determining the green-fluorescent punctate pattern were observed under fluorescence microscopy. The percentage of green fluorescence intensity was assessed by image analysis to determine the degree of autophagy.

**Transcriptional analyses**

***RNA extraction and Real-Time PCR experiments***

Total RNA was extracted from hPEACs (*n* = 3 independent experiments) cell culture by a dedicated kit (RNeasy Plus Micro Kit, Qiagen SpA, Milano, Italy) optimized to purify total RNA from small amounts of cells (<5*10^5^) as reported in our previous work (22). Briefly, after re-suspensions, hPAECs are first lysed and homogenized in highly denaturing guanidine-isothiocyanate-containing buffer RLT Plus, which immediately inactivates RNases to ensure isolation of intact RNA. Then, the samples were passed through a gDNA Eliminator spin column. Ethanol was added to the flow-through to provide appropriate binding conditions for RNA, and then the samples were applied to a silica-based membrane (RNeasy MinElute spin column) and speeded on microcentrifuge at 12000 RPM for 30 seconds; specific buffers allowed RNA to bind to the RNeasy silica-membrane and contaminants were efficiently washed away. High-quality RNA was then eluted in RNAse-free water without additional DNase digestion.

The total RNA sample concentration was determined by measuring the absorbance at 260 and 280 nm (NanoDrop Thermofisher, Waltham, MA, USA) and calculated using the Beer-Lambert law (expected values between 1.8 and 2.1). The total RNA was then reverse-transcribed in first-strand cDNA by Mir-X miRNA first stand syntesis Kit (Takara BIO, USA). Gene expressions were determined by Real-Time PCR in the Bio-Rad C1000 TM thermal cycler (CFX-96 Real-Time PCR detection systems, Bio-Rad) and monitored with EvaGreen (SsoFAST EvaGreen Supermix, Bio-Rad). Mature miRNA sequences used as forward primer for miRNAs detection were downloaded from the miRBase database (www.mirbase. org) and synthesized by the Merck-Sigma company (Milan, I) (**Online** **Table 1**). To assess product specificity, amplicons were systematically checked by melting curve analysis. Melting curves were generated from 65°C to 95°C with increments of 0.5°C/cycle. The MIQE Guidelines (23) for a correct and reproducible Real-Time PCR experiment were followed (**Online** **Table 1**).
