# Supplementary material for: Ambrisentan Retains Its Pro‐Autophagic Activity on Human Pulmonary Artery Endothelial Cells Exposed to Hypoxia in an In Vitro Model Mimicking Diabetes
Source: J Cell Mol Med. 2025 Apr 9;29(7):e70528. doi: 10.1111/jcmm.70528 (PMC11982177; doi:10.1111/jcmm.70528)

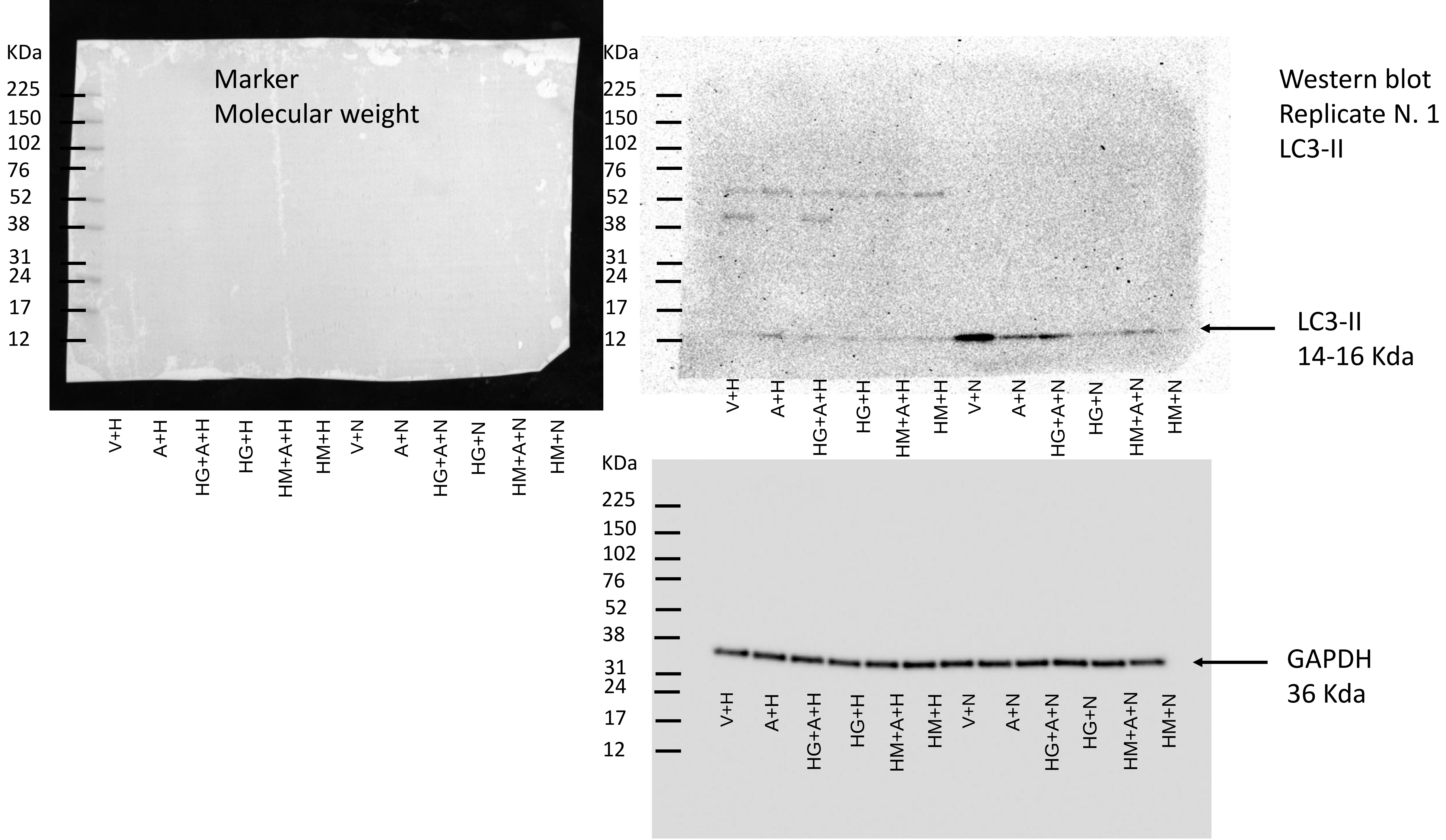
KDa
225
150
102
76
52
38
31
24
17
12
Marker
Molecular weight
V+H
A+H
HG+A+H
HG+H
HM+A+H
HM+H
V+N
A+N
HG+A+N
HG+N
HM+A+N
HM+N
Western blot
Replicate N. 1
LC3-II
LC3-II
14-16 Kda
GAPDH
36 Kda

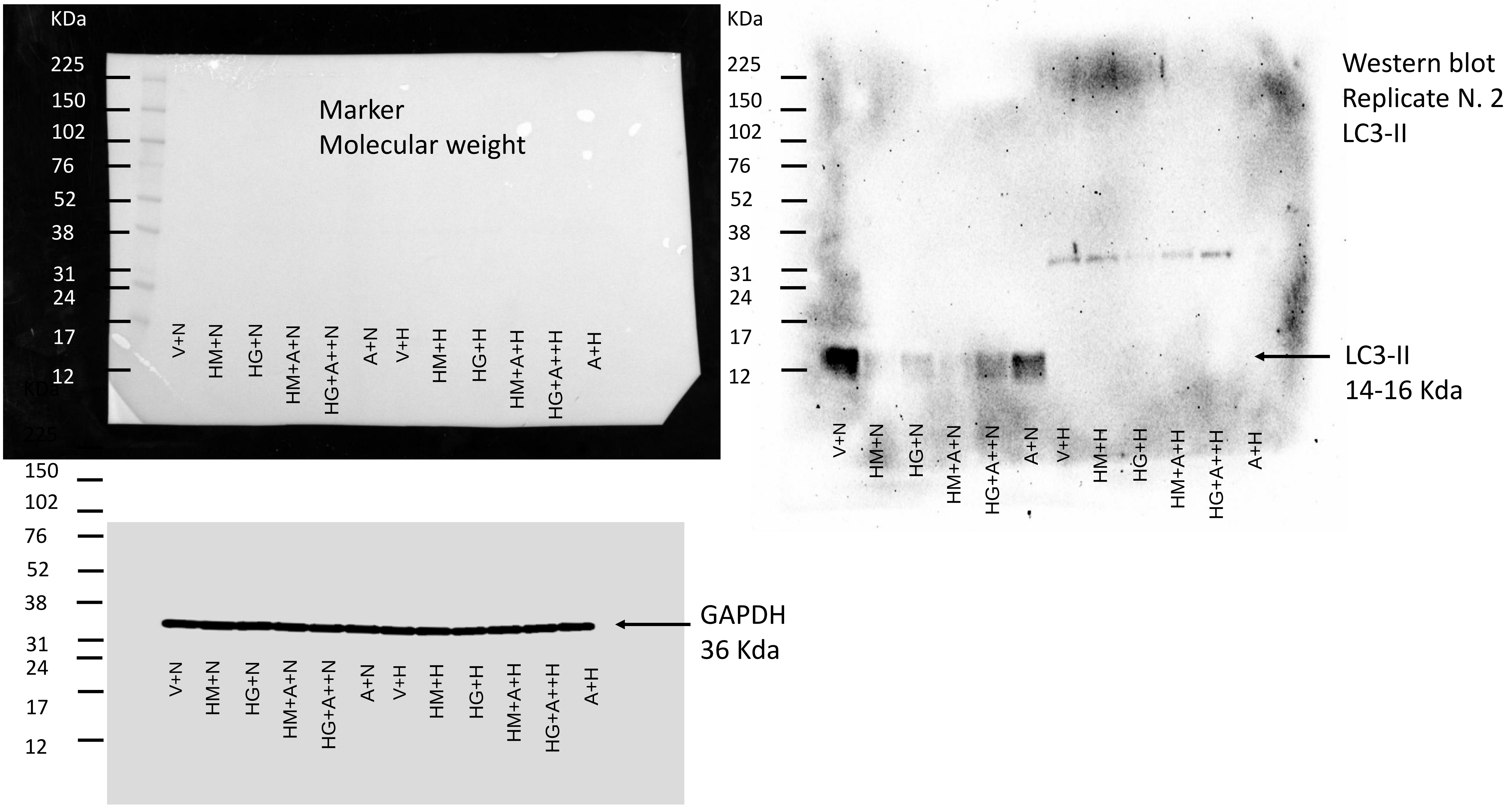
KDa
225
150
102
76
52
38
31
24
17
12
Marker
Molecular weight
V+N HM+N HG+N HM+A+N HG+A++N A+N V+H HM+H HG+H HM+A+H HG+A++H A+H
KDa
225
150
102
76
52
38
31
24
17
12
Western blot
Replicate N. 2
LC3-II
LC3-II
14-16 Kda
V+N HM+N HG+N HM+A+N HG+A++N A+N V+H HM+H HG+H HM+A+H HG+A++H A+H
KDa
225
150
102
76
52
38
31
24
17
12
GAPDH
36 Kda
V+N HM+N HG+N HM+A+N HG+A++N A+N V+H HM+H HG+H HM+A+H HG+A++H A+H

Western blot
Replicate N. 3
LC3-II

Marker
Molecular weight

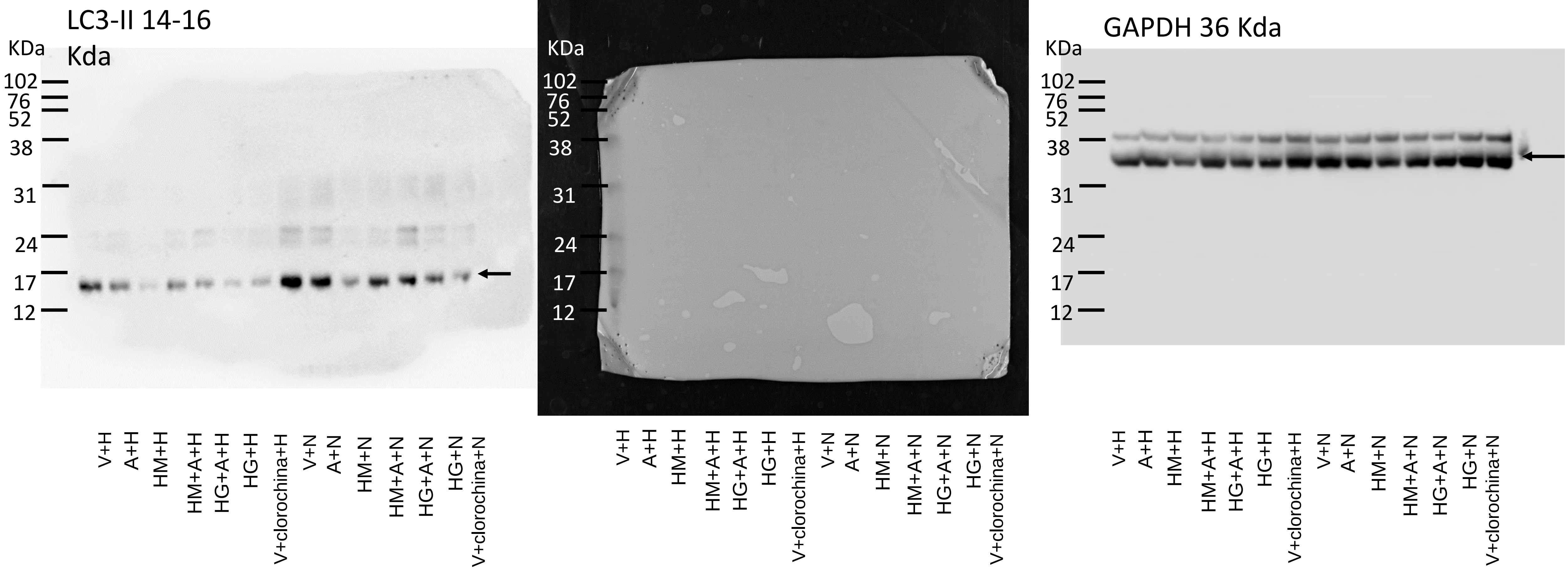

Western blot Replicate N. 1 Cleaved Caspase-3

Early exposure

late exposure

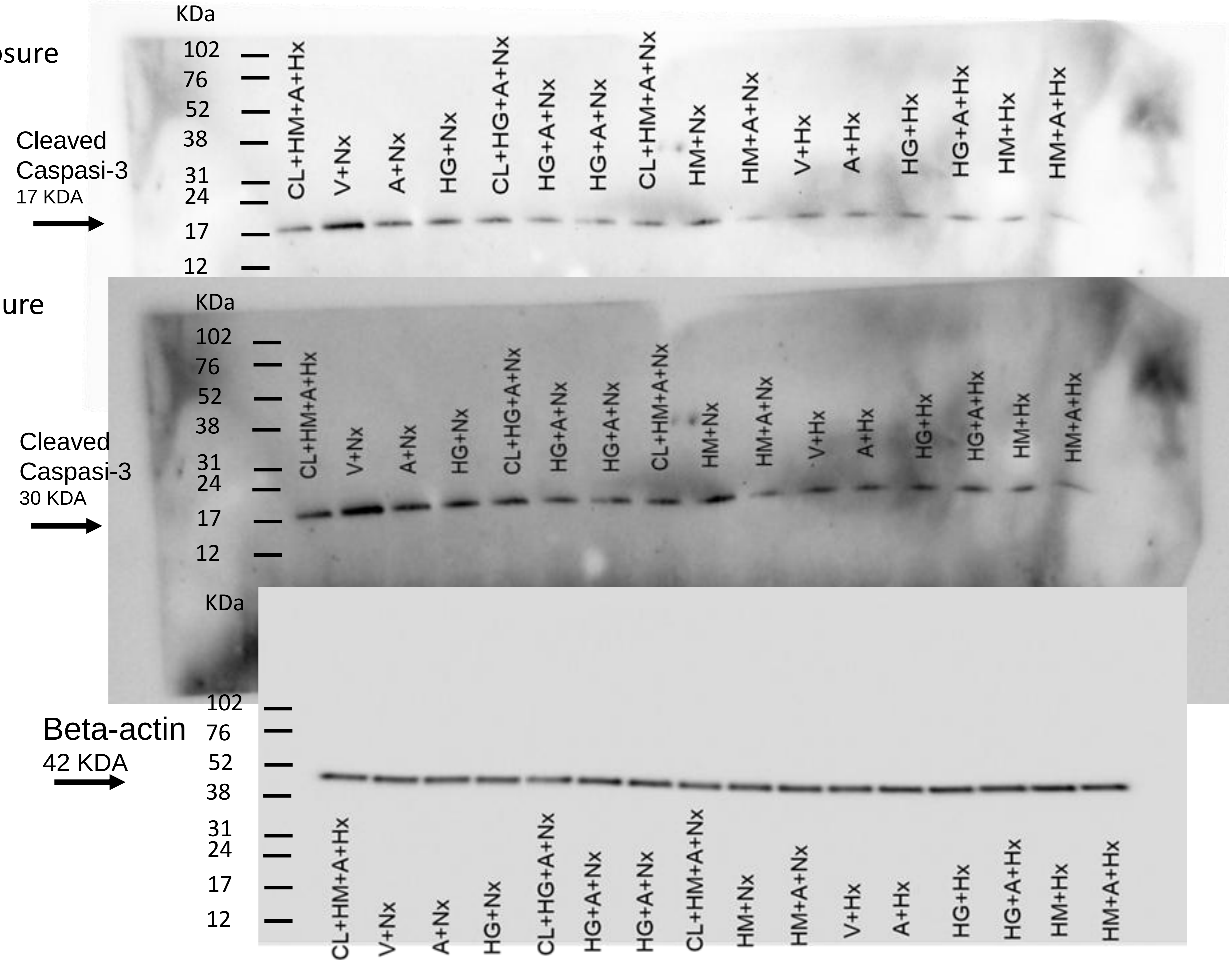

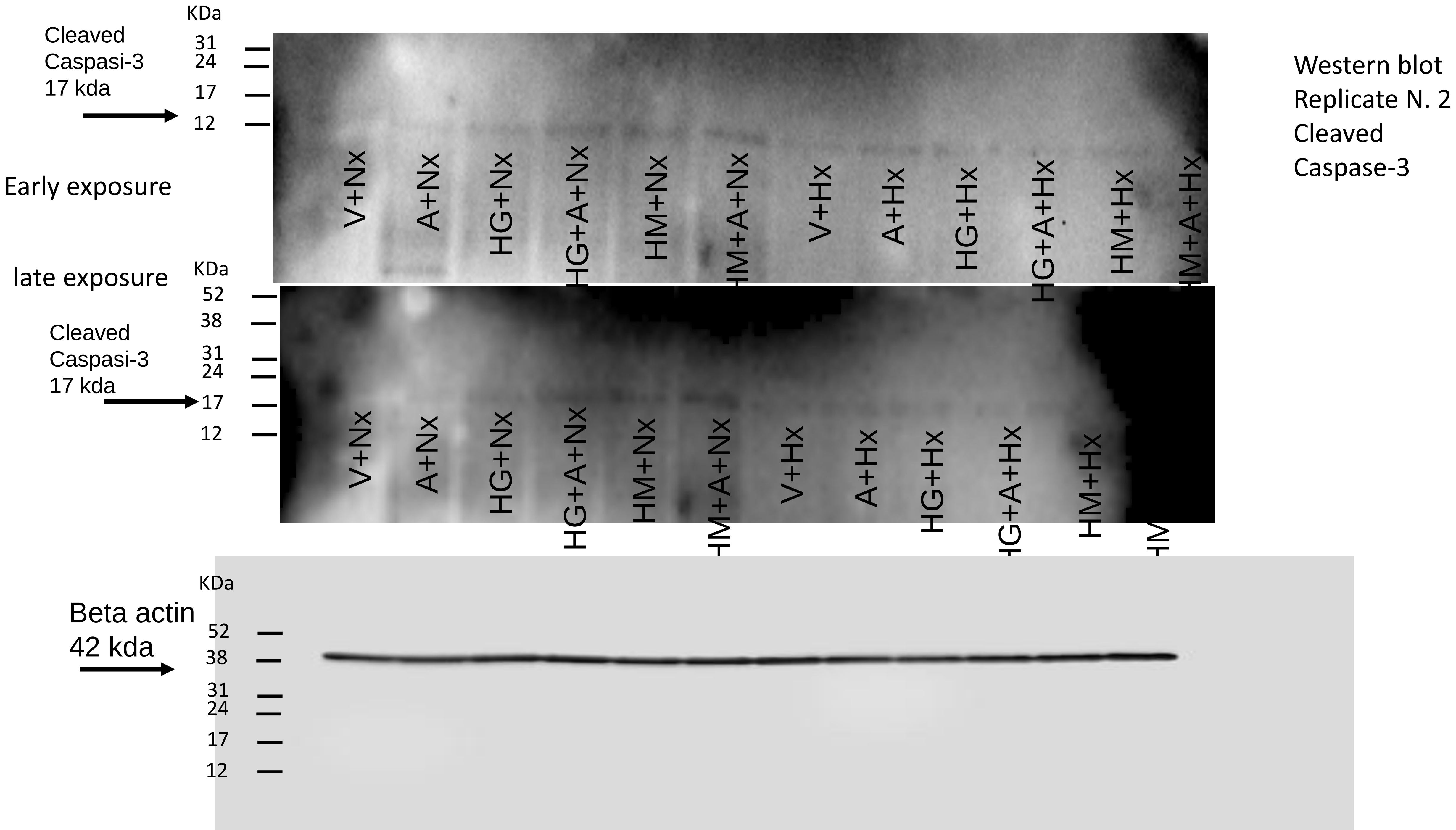

Western blot
Replicate N. 2
Cleaved
Caspase-3
Early exposure
Cleaved
Caspasi-3
17 kda
KDa
31
24
17
12
V+Nx
A+Nx
HG+Nx
HG+A+Nx
HM+Nx
HM+A+Nx
V+Hx
A+Hx
HG+Hx
HG+A+Hx
HM+Hx
HM+A+Hx
late exposure
Cleaved
Caspasi-3
17 kda
KDa
52
38
31
24
17
12
V+Nx
A+Nx
HG+Nx
HG+A+Nx
HM+Nx
HM+A+Nx
V+Hx
A+Hx
HG+Hx
HG+A+Hx
HM+Hx
Beta actin
42 kda
KDa
52
38
31
24
17
12

Western blot
Replicate N. 3
Cleaved
Caspase-3

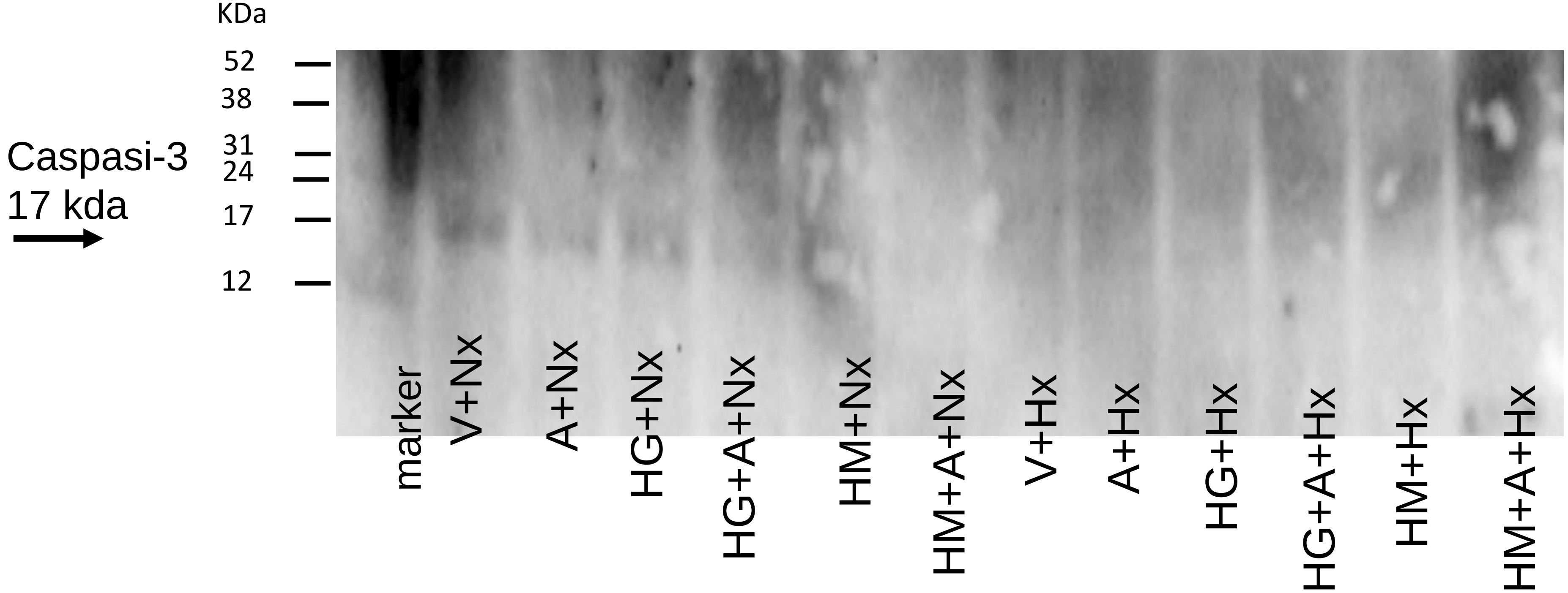

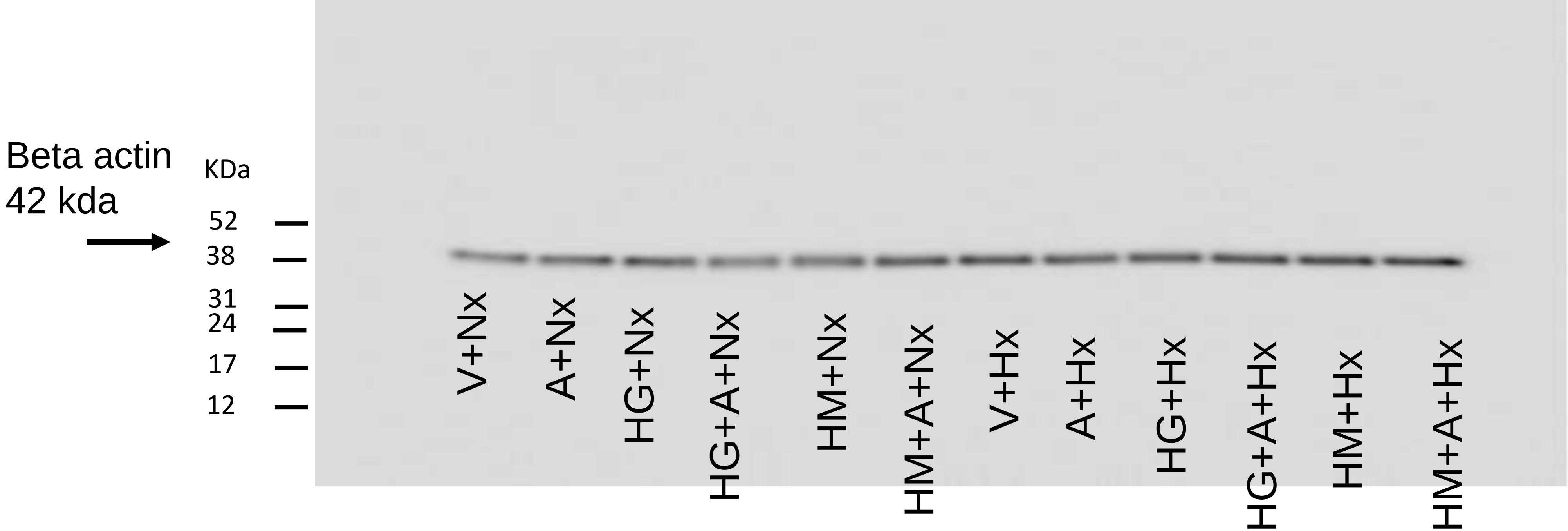

Supplement: Supplementary file 5 — Data S3. [file JCMM-29-e70528-s003.pdf]
